# Polycyclic aromatic hydrocarbons and polychlorinated biphenyls in tissue of mangrove *Rhizophora mangle *from the Colombian Caribbean

**DOI:** 10.1007/s10661-026-15031-8

**Published:** 2026-02-16

**Authors:** Laura García-Meléndez, Nicolás Santos-Vásquez, Néstor Hernando Campos-Campos, Adolfo Sanjuan-Muñoz, Néstor Javier Mancera-Rodríguez, Mario Monroy

**Affiliations:** 1 Departamento de Ciencias Forestales, Facultad de Ciencias Agrarias, Universidad Nacional de Colombia, Carrera 65 No. 59 A - 110, Bloque 14, Oficinas de Posgrado 4.º Piso, Sede Medellín, Medellín, Colombia; 2 Área de Ciencias Biológicas y Ambientales, Facultad de Ciencias Naturales E Ingeniería, Universidad de Bogotá Jorge Tadeo Lozano, Sede Santa Marta., Edificio Mundo Marino. El Rodadero., Carrera 2 # 11-68, Santa Marta, Colombia; 3 Instituto de Estudios en Ciencias del Mar (Cecimar), Universidad Nacional de Colombia Sede Caribe, Santa Marta, Colombia; 4 Departamento de Biología, Facultad de Ciencias, Universidad Nacional de Colombia, Sede Bogotá, Carrera 45 No. 26-85, Bogotá, Colombia

**Keywords:** Carcinogenic compounds, Ciénaga Grande de Santa Marta, Cispatá Bay, Coastal ecosystems, Organic pollutants

## Abstract

The expansion of human activities has led to organic compound contamination. These compounds have adverse ecosystem effects, yet research on their bioaccumulation is insufficient globally. This study aimed to (i) determine PAH and PCB concentration levels in *Rhizophora mangle* root tissue, and (ii) evaluate seasonal variations in PAH and PCB levels between rainy and dry seasons in two estuarine areas: the Ciénaga Grande de Santa Marta (CGSM) and Cispatá Bay (CIS). Samples from three places per area, collected in September 2022 and April 2023, were analyzed by gas chromatography-mass spectrometry. Results revealed that PAH concentrations were highest during the rainy season, in the Cispatá Bay, with an average of 180.5 ± 21.4 ng g^−1^. In contrast, PCB concentrations peaked during the dry season, in CGSM, with an average of 10.7 ± 11.6 ng g^−1^. A significant positive correlation was observed between silt and total PAH concentrations during the rainy season. Conversely, total PCBs showed a significant negative correlation with pH and fine sediment during the rainy season, and with coarse sediment during the dry season. Using the Biota-Sediment Accumulation Factor (BSAF), it was found that root tissues can only accumulate small amounts of PAHs from sediments. Additionally, the presence of albino mangrove individuals in the Cispatá area suggests potential mutagenic stress. These findings underscore the urgent need to mitigate contamination and standardize monitoring protocols to protect biodiversity in the Colombian coastal ecosystems.

## Introduction

Environmental pollution is a global issue of critical concern, threatening global health and biodiversity at multiple levels (Bashir et al., 2020; Mojiri et al., 2019; Ontiveros-Cuadras et al., 2019). Among the most hazardous contaminants, organic pollutants (OPs) represent a significant risk primarily in aquatic ecosystems (Bayen, 2012; El-Amin Bashir et al., 2017; Veldkornet et al., 2020). These chemical compounds encompass a wide variety of carbon-based substances, whose environmental impact is closely tied to their persistence and toxicity (Maletić et al., 2019; Mukherjee et al., 2022; Ontiveros-Cuadras et al., 2019). OPs are known to cause endocrine disruption, immunotoxicity, neurotoxicity, and carcinogenic potential (USEPA, 2013; Pino et al., 2016; Billah et al., 2022). Due to hydrodynamic processes and sediment dynamics, these pollutants tend to accumulate in estuarine and mangrove areas, where conditions such as low salinity and high sediment retention facilitate their bioavailability (Burgos-Núñez et al., 2017; Qiu et al., 2018; Robin & Marchand, 2022).

Mangrove forests are key coastal ecosystems located in tropical and subtropical intertidal zones, acting as natural buffers and providing essential habitats for a wide range of species (Awuku-Sowah et al., 2022; Feller et al., 2010; Huxham et al., 2017). These systems possess unique physiological adaptations such as salt-excreting glands and specialized roots, which allow them to thrive in brackish and flooded environments (Kathiresan & Bingham, 2001; Naskar & Palit, 2015). One of their most important ecosystem services is their role in improving biogeochemical cycling, as they effectively filter and retain pollutants and sediments, thereby enhancing water quality and supporting ecological resilience (Qiu et al., 2018; Robin & Marchand, 2022).

Mangroves should also be considered crucial allies in the fight against global warming, representing one of the most efficient "blue carbon" ecosystems on the planet (Alongi, 2020). The majority of this carbon is not stored in trees themselves but rather remains sequestered for centuries or even millennia in the deep, waterlogged soils (Kida & Fujitake, 2020). This long-term storage capacity makes mangrove conservation a crucial natural solution for mitigating climate change (Song et al., 2023; Taillardat et al., 2018).

Despite their ecological importance, mangrove ecosystems are increasingly threatened by contamination from organic pollutants. These compounds accumulate in different environmental compartments, including water, sediments, and the tissues of organisms (Cahyaningsih et al., 2022; Dai et al., 2022; Reizer et al., 2022). Physicochemical variables such as pH, grain size, and organic matter content influence the distribution and persistence of OPs, potentially leading to bioaccumulation in mangrove-associated organisms (Ambade et al., 2022; Robin & Marchand, 2022). In this context, mangroves, as unique forest ecosystems worldwide, play a crucial role in maintaining ecological balance (Awuku-Sowah et al., 2022; Cahyaningsih et al., 2022; Huxham et al., 2017).

Two major classes of OPs in coastal ecosystems are polycyclic aromatic hydrocarbons (PAHs) and polychlorinated biphenyls (PCBs). PAHs are derived from both natural sources, such as forest fires, and anthropogenic activities, including fossil fuel combustion and hydrocarbon spills (Abdel-Shafy & Mansour, 2016; Faroon & Ruiz, 2016; USEPA, 2023). PCBs, widely used in industrial applications, can reach aquatic environments through leachates, atmospheric deposition, and runoff from urban and industrial areas, particularly from electrical equipment containing thermal insulators and as a result of incineration processes (Bayen, 2012; USEPA, 2013; Robin & Marchand, 2022).

The environmental behavior of these compounds is largely determined by their molecular structure. PAHs are categorized by molecular weight: low molecular weight (LMW) PAHs (2–3 aromatic rings) are more soluble and mobile, whereas high molecular weight (HMW) PAHs (4–6 aromatic rings) are more hydrophobic and tend to accumulate in lipid-rich tissues (Abdel-Shafy & Mansour, 2016; Faroon & Ruiz, 2016; Meng et al., 2019; Wang et al., 2014). PCB, composed of benzene rings with varying chlorine substitutions, are classified as either dioxin-like PCBs (dl-PCBs) or non-dioxin-like PCBs (ndl-PCBs), with the former being more toxic (Vane et al., 2009; Pemberthy et al., 2016; Simhadri et al., 2020; Ávila et al., 2022). Seven PCB congeners are commonly used as environmental indicators for monitoring contamination: PCB 28, PCB 52, PCB 101, and PCB 118, which are categorized as light PCBs (comprising tri-, tetra-, and penta-chlorinated compounds), while PCB 138, PCB 153, and PCB 180 are classified as heavy PCBs (including hexa-, hepta-, and octa-chlorinated congeners) (Alegría et al., 2016; Mbusnum et al., 2020). These seven PCB congeners were selected as indicators, based on international scientific consensus (led by ICES) due to their abundance and environmental persistence (Fernandes et al., 2010). The Dutch list is derived from the first German congener-specific report (Koopman-Esseboom et al., 1994; Schantz et al., 2003), which examined the transfer of these environmentally available compounds from nursing mothers to their infants.

Mangrove plants take up PAHs and PCBs primarily through root adsorption and absorption, with further translocation to other plant tissues (Billah et al., 2022; Robin & Marchand, 2022; Wang et al., 2010). PAHs can also be absorbed atmospherically through the waxy stomatal coating of mangrove leaves (Robin & Marchand, 2022; Wang et al., 2010). Accumulation patterns may vary among species and even between plant organs, with leaves often acting as the primary site of contaminant accumulation due to their exposure to air (Billah et al., 2022; Qiu et al., 2018). The uptake of these compounds may cause structural and functional damage in roots, including lipid membrane disruption and disorganization of cellular organelles, potentially leading to cell death (Naidoo & Naidoo, 2016, 2017, 2018; Zaalishvili et al., 2002). Other documented effects include signs of foliar stress, irregular stomatal behavior, and chlorophyll deficiencies associated with saturation by LMW PAHs (Robin & Marchand, 2022; Veldkornet et al., 2020). Additionally, they may impair physiological processes such as growth rate and photosynthesis, with reported root concentrations reaching 10,280 μg kg^−1^ of total PAHs (ƩPAHs) and 60 μg kg^−1^ in leaves under laboratory conditions (El-Amin Bashir et al., 2017; Naidoo & Naidoo, 2016; Veldkornet et al., 2020).

Due to its direct interaction with sediments, *Rhizophora mangle* (red mangrove) is particularly vulnerable to contamination (Proffitt & Travis, 2005; Robin & Marchand, 2022). This species is widely distributed along tropical and subtropical coastlines from West Africa to the Americas, including the Caribbean and the Colombian Pacific coast (DeYoe et al., 2020; Sánchez et al., 2021). As a pioneer species, *R. mangle* exhibits two growth forms (tall trees and shrubs), and develops distinctive stilt roots, which provide stability and facilitate gas exchange in waterlogged environments (DeYoe et al., 2020; Naidoo & Naidoo, 2018).

In Colombia, a few studies have addressed the presence of compounds associated with chlorinated fertilizers and their seasonal variations in tissues of *R. mangle* and *Avicennia germinans* and *Crassostrea rhizophorae* (Espinosa et al., 1995; Aguirre-Rubí, et al., 2018; Angulo-Cuero et al., 2021). The presence of PAHs in these ecosystems is expected, given their multiple anthropogenic sources and previous reports documenting their occurrence in other environmental compartments such as water and sediments (Caballero-Gallardo et al., 2015; Mejía-Monterroza, 2015; Burgos-Núñez et al., 2017; Nunes et al., 2021; García et al., *on press*). Despite this, to date, no specific studies have directly assessed the concentrations of these compounds in mangrove tissues. This represents a significant knowledge gap, considering the ecological importance of mangrove in contaminant retention and biogeochemical cycling. Therefore, the aims of this study were: (i) to quantify the concentration of PAHs and PCBs in the root tissue of *R. mangle*, and (ii) to assess the seasonal variations in these concentrations between the rainy and dry seasons in two estuarine zones of the Colombian Caribbean. This research provides essential baseline information on contaminant accumulation in mangrove systems highlighting the need to implement targeted mitigation and conservation strategies to protect these critical coastal ecosystems.

## Materials and methods

### *Study area*

**Fig. 1:**
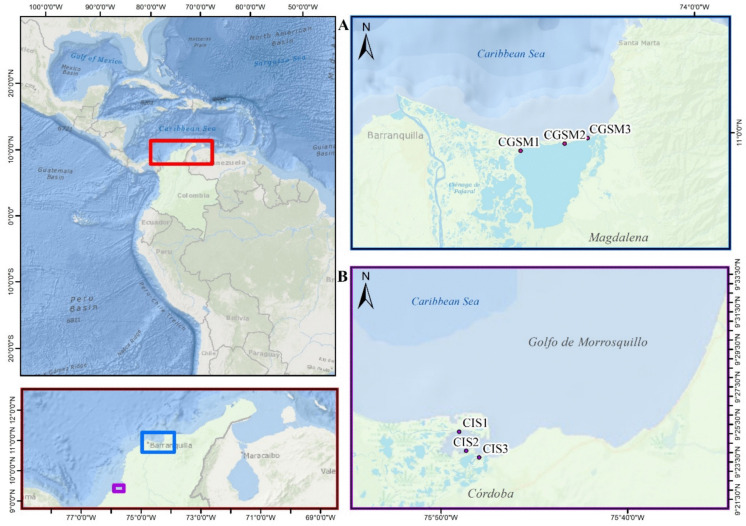
Map showing the sampling points in two estuarine areas of the Colombian Caribbean, highlighted within the red box. The blue rectangle (**A**) identifies the location of the Ciénaga Grande de Santa Marta (CGSM), while the purple rectangle (**B**) indicates the Cispatá Bay (CIS)

This study was conducted in two estuarine zones along the Colombian Caribbean coast, located at the upper western edge of South America (Fig. 1): the Ciénaga Grande de Santa Marta (CGSM), and Cispatá Bay (CIS). The former is influenced by discharges from the Magdalena River and is characterized by a bimodal climatic pattern, with two dry seasons (from December to April and June to July) and two rainy seasons (May and September to November) (Ricaurte-Villota & Bastidas Salamanca, 2017; Caballero-Gallardo et al., 2021; Rodríguez-Grimón et al., 2021). In contrast, CIS is primarily influenced by the discharge from the Sinú River and exhibits a unimodal climatic regime, with a single dry season from late January to mid-March and a well-defined rainy season from mid-March to early January, peaking in intensity during October (Mejía-Monterroza, 2015; Ricaurte-Villota & Bastidas Salamanca, 2017).

It is important to characterize these historically contaminated regions. Specifically, the Ciénaga Grande de Santa Marta (CGSM), near Cartagena Bay, is exposed to multiple contamination vectors, including historical discharges from chlorine-based industrial activities, agricultural runoff, and pesticide spills from the Magdalena River basin (Espinosa-Díaz et al., 2021). Similarly, the Gulf of Morrosquillo faces long-term contamination due to oil spills and waste accumulation. Additionally, a critical factor in these areas is the risk of recontamination caused by sediment resuspension during activities such as dredging (Alegría et al., 2016; Garcés-Ordóñez et al., 2019; Hosoda et al., 2014).

The Colombian Caribbean coast is under to multiple intensive anthropogenic activities, including livestock farming, agriculture, tourism, and hydrocarbon extraction, many of which occur in proximity to major river basins and offshore areas (DANE, 2022; ANH, 2023). Major port cities near the estuarine zones, such as Coveñas, Cartagena, Barranquilla, and Santa Marta, contribute to the input of wastewater and other pollutants into adjacent coastal ecosystems (INVEMAR, 2014). The Magdalena and Sinú rivers play a significant role in the transport of water and sediments into these ecosystems.

The Magdalena River spans approximately 1,540 km, respectively, with a drainage basin of 277,440 km^2^ and an average water discharge of 7,232 m^3^s^−1^, transporting and estimated 390.869*10^3^ td^−1^ of suspended sediments (Restrepo-López et al., 2015). The Sinú River, in contrast, is approximately 300 km long, with a 14,700 km^2^ basin, an average discharge of 383 m^3^s^−1^ and a sediment load of 8.620*10^3^ td^−1^ (Restrepo-López et al., 2015). The convergence of natural hydrological processes and human-induced pressures in these areas likely influences the presence and transport of pollutants compounds, including PAHs and PCBs, within the mangrove ecosystem.

### *Sampling design*

During the preliminary sampling in November 2021 (rainy season) and April 2022 (dry season), sediment subsamples were collected using a 0.05 m^2^ Van Veen dredge to determine PAH concentrations. All samples were refrigerated and stored in portable coolers at 0 °C until they were transported to the facilities of the University of Córdoba, Colombia (García et al., on press).

Root tissue samples of *R. mangle* were collected during two distinct climatic periods: the rainy season (September 2022) and the dry season (April 2023), with average rainfall of 6.14 + 11.83 mm in Magdalena and 6.10 + 12.90 mm in Sinú (calculated for the months of July, August, and September; JAS), 0.73 + 6.04 mm in Magdalena and 0.43 + 3.59 mm in Sinú (calculated for the months of February, March, and April; FMA), under a La Niña ENSO regime (DHIME, 2025; NOAA, 2025). Sampling was conducted at two estuaries along the Colombian Caribbean coast, Ciénaga Grande de Santa Marta (CGSM) and the Cispatá Bay (CIS), with three sampling points per location.

At each point, three replicate samples were compiled at each point. All activities were carried out under a framework agreement with the Universidad de Bogotá Jorge Tadeo Lozano, in compliance with environmental permit ANLA Resolution No. 00213, issued on January 28, 2021. At each sampling site, root tissue was collected from the rhizosphere of the mangrove trees exhibiting well-developed root systems. Approximately 200 g of rhizosphere material were extracted per sample unit and combined to create one composite sample per point. Samples were wrapped in aluminum foil, sealed in Ziploc® bags, and stored on ice (0 °C) during transport to preserve their integrity, following the protocol described by Chowdhury et al. (2021).

In situ measurements of surface-level physicochemical parameters were conducted at each sampling point. Parameters including temperature (Temp, °C), salinity (Sal), conductivity (Cond, mS cm^−1^), pH, dissolved oxygen (DO, mg L^−1^), and oxygen saturation (OS, %) were taken using YSI pro1030 and YSI pro20 multiparameter probes. Sediment samples were also collected for granulometric analysis. Approximately 100 g of sediment from each point was oven-dried at 110 °C until fully dehydrated and then sieved through a series of graduated meshes. Based on grain size, samples were classified into three fractions: coarse sand (Csand,1 mm to 0.250 mm), fine sand (Fsand, 0.180 mm to 0.063 mm), and silt (< 0.063 mm), following the methodology of Kenny and Sotheran (2013).

Additional laboratory analyses were conducted to determine organic matter (OM) content and redox potential. OM was quantified by combusting 5 g of dried sediment in a muffle furnace at 550 °C for 5 h. The content was expressed as a percentage of weight loss, using the formula: (g OM × g^−1^ sample processed) × 100% (Kenny & Sotheran, 2013). For redox potential, 25 g of dried sediment were incubated at 40 °C for 24 h, homogenized in 50 mL of distilled water, and stirred for 30 min. Measurements were taken at 5 and 10 min using the YSI Pro-1030 multiparameter probe under constant temperature (~ 25 °C), and expressed in millivolts (mV) (Aldridge & Ganf, 2003).

### *Chemical analysis for PAHs and PCBs determination*

The determination of PAHs and PCBs in plant tissue samples was performed externally at the Diagnostic and Contamination Control Laboratory (GDCON) of the University of Antioquia (Colombia), an ISO/IEC 17025-accredited facility. The analysis followed the U.S. Environmental Protection Agency (USEPA) methods 3550 C (ultrasonic extraction; USEPA, 2007a) and 8082 A (gas chromatography for PAHs and PCBs, USEPA, 2007b).

Analyte quantification was performed using an external calibration curve prepared from a Certified Reference Material (CRM) consisting of standard mixtures of PAHs 10 mg L^−1^/1.0 mg L^−1^ and PCBs 10 mg L^−1^/1.0 mg L^−1^ (batches S2303281157 and S2303281158), ensuring full traceability of all measurements. To ensure the reliability of the results, a rigorous quality control protocol was implemented: a method blank was analyzed in each batch to verify the absence of contamination (< 0.5 * LC); the accuracy of the method was evaluated with an analytical control standard of 100 µg L^−1^ (PAH), and 50 µg L^−1^ (PCB), prepared from the same MRC; and additionally, the effect of the matrix was evaluated by analyzing triplicate replicates of a real sample (code 23–6861-3) enriched with 70 µg L^−1^ (PAH), and 30 µg L^−1^ (PCB), to validate the performance and recovery of the method. Method quantification limits were 40 µg kg^−1^ (0.04 mg kg^−1^) for PAHs and 10.0 µg kg^−1^ (0.01 mg kg^−1^) for PCBs in soils.

For sample preparation, 1.0 g of dried plant tissue was mixed with 2.0 g of anhydrous sodium sulfate in a plastic centrifuge tube. A solvent mixture of n-hexane–acetone was added, and the sample underwent ultrasonic extraction. Following extraction, the mixture was centrifugated at 6000 rpm for 3 min. The resulting supernatant was collected and transferred into a vial to obtain a final volume of 25 mL. The extract was subsequently concentrated under vacuum and further evaporated to a final volume of approximately 2–3 mL. The concentrated solution was then transferred, homogenized, measured, sealed and loaded into an autosampler for instrumental analysis. Chromatographic analysis was performed using an Agilent 7890 A gas chromatograph coupled to a 5975 C mass selective detector (GC–MS). A 2.0 µL aliquot of each sample was injected into the system. Results were quantified and expressed as nanograms of analyte per gram of dry plant tissue (ng g^−1^dw).

### *Data analysis*

To explore correlations among physicochemical variables, and to reduce dimensionality among explanatory variables, a Principal Component Analysis (PCA) was performed using PAST software (version 4.03). Spearman correlation analyses were conducted to assess relationships between surface-level physicochemical variables (temperature, salinity, pH, and oxygen saturation), sediment characteristics (OM, redox potential, percentage of coarse sand, fine sand, and silt), and the concentrations of total LMW and HMW PAHs, as well as total PCBs. These analyses were carried out separately for the rainy and dry seasons using Rstudio software (Version 2024.04.2 + 764) with the support of various packages: gridExtra, gapminder, dplyr, ggplot2, tidyverse, corrplot, dendextend, NbClust, factoextra, and skimr. Statistical significance was assessed at three thresholds: p-values < 0.05, < 0.01, and < 0.001.

For each sampling point in both CGSM and CIS, the proportion of carcinogenic PAH compounds (Σ PAH^C^) relative to the total PAH concentration (Σ PAH), was calculated and expressed as a percentage.

To evaluate the accumulation of PAHs in *R. mangle* roots from sedimentary sources, the Biota-Sediment Accumulation Factor (BSAF) was calculated using the following equation:

$$BSAF=\frac{{C}_{b}}{{C}_{s}}$$

where C_b_ represents the concentration of a specific PAH in root tissue, and C_s_ the corresponding concentration in sediments. Only PAH concentrations were considered for this calculation. Sediment data were obtained from previously published work (García et al., on press), which included samples from both CGSM and CIS. BSAF values greater than 1 indicate bioaccumulation of persistent organic pollutants (POPs) by the organism (Kwok et al., 2013; Qiu et al., 2019; Zhu et al., 2014). Based on BSAF values, the accumulation capacity of *R. mangle* was categorized as follows: macro concentrator (BSAF > 2), micro concentrator (1 < BSAF < 2), or de-concentrator (BSAF < 1) (Qiu et al., 2019).

## Results

### *PAHconcentrations*

The highest PAH concentrations in mangrove roots were recorded in the Cispatá Bay (CIS) during the rainy season, with a mean of 180.45 ± 21.4 ng g^−1^ dry weight (dw), ranging from 156.8 to 198.5 ng g^−1^. These concentrations were predominantly composed of HMW compounds, with carcinogenic PAHs such as BaA, BaP, BbF, BkF, and Ch accounting for 64.0% of the total (see Table 1). In contrast, lower PAH concentrations were observed in the Ciénaga Grande de Santa Marta (CGSM) during the same season, averaging 86.9 ± 31.3 ng g^−1^ dw (ranging from 55.9 to 118.4 ng g^−1^). The PAH profile in CGSM reflected a mixture of LMW compounds (e.g., Pn and An) and HMW compounds (e.g., Ch, BbF, BkF), with carcinogenic PAHs comprising 34.8% of the total (Table 1).

**Table 1:** Average PAH concentrations (in ng g^−1^ dw) and standard deviation in *Rhizophora mangle* root tissues, by sampling location, season (rainy and dry), and contaminant compounds in Ciénaga Grande de Santa Marta (CGSM) and the Cispatá Bay (CIS), with three sampling points in each one. The data include low molecular weight (LMW) and high molecular weight (HMW) PAHs, total PAH concentrations (ΣPAH), and. total carcinogenic PAHs (PAH^C^). Abbreviations: Na-naphthalene, Ayl-acenaphthylene, Aen-acenaphthylene, Pn-phenanthrene, An-anthracene, Fl-fluoranthene, Py-pyrene, BaA-benzo[a]anthracene, Ch-chrysene, BbF-benzo[b]fluoranthene, BkF-benzo[k]fluoranthene, BaP-benzo[a]pyrene. ΣPAH_sediment_: Total PAH concentrations in sediment at corresponding sampling locations. ND: not detected (below detection limit). * Compounds classified as carcinogenic PAHs. BSAF: Biota-Sediment Accumulation Factor

Compound	Rainy		Dry	
	CIS	CGSM	CIS	CGSM
Na	8.9 ± 4.8	4.1 ± 1.4	4.1 ± 0.6	2.8 ± 2.4
Ayl	1.9 ± 3.2	1.7 ± 2.9	4.0 ± 3.6	ND
Aen	ND	ND	1.6 + 2.7	ND
Pn	20.5 ± 4.6	23.9 ± 5.8	22.9 ± 6.3	23.2 ± 7.9
An	12.6 ± 2.4	11.7 ± 0.9	9.8 ± 9.2	ND
Fl	14.6 ± 2	13.9 ± 1.4	13.7 ± 1.9	13.7 ± 1.7
Py	6.6 ± 2.6	6.3 ± 2	5.1 ± 1.9	5.5 ± 2
BaA*	34.2 ± 2.5	ND	13.3 + 22.9	10.6 ± 18.4
Ch*	20.3 ± 3.5	11.8 ± 10.2	12.1 ± 10.5	6.3 ± 10.9
BbF*	26.4 ± 6.5	6.7 ± 11.6	6.1 ± 10.6	11.8 ± 10.5
BkF*	23.7 ± 2.6	6.9 ± 11.87	ND	7.0 ± 12.2
BaP*	10.9 ± 18.8	ND	ND	ND
LMW	43.9 ± 7.1	41.3 ± 4.9	42.4 ± 14.5	25.9 ± 10
HMW	136.6 ± 21.2	45.6 ± 32.7	50.3 ± 14.1	54.8 ± 24.2
ΣPAH	180.5 ± 21.4	86.9 ± 31.3	92.7 ± 25.7	80.8 ± 30.4
PAH^C^	115.5 ± 19.7	25.3 ± 29.9	31.5 ± 12.1	35.7 ± 22.6
% PAH^C^	63.9 ± 5.9	23.2 ± 24.8	33.7 ± 9.9	41.6 ± 13.5
ΣPAH_sediment_	263.8 ± 140.3	110.1 ± 5.2	1,599.5 ± 1,293.4	174.2 ± 43.2
BSAF	0.8 ± 0.6	0.8 ± 0.3	0.2 ± 0.2	0.5 ± 0.1

During the dry season, PAH concentrations decreased in both locations. CIS recorded an average of 92.7 ± 25.7 ng g^−1^ (ranging from 72.4 to 121.6 ng g^−1^), with both LMW (e.g., Pn, An), and HMW (e.g., BaA, Ch, Fl) compounds present. Carcinogenic PAHs account for 33.7% of the total. Meanwhile, CGSM showed a slightly lower mean concentration of 80.76 ± 30.42 ng g^−1^ (ranging from 46.6 to 104.9 ng g^−1^), but with a predominance of HMW compounds (e.g., BaA, BbF, Ch, Fl) and PAH^C^, representing 41.6% of the total (Table 3).

**Fig. 2:**
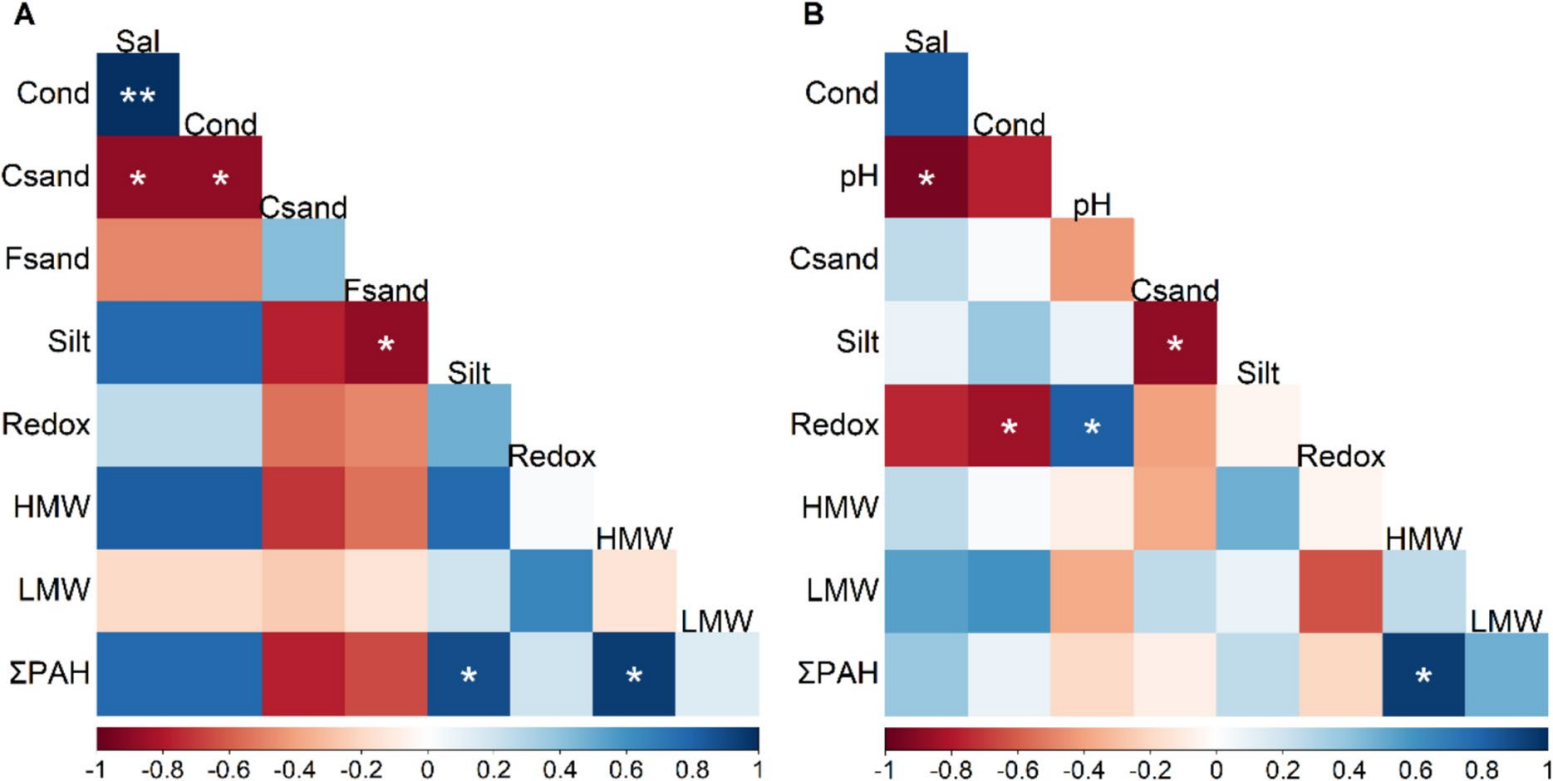
Spearman correlation matrices between environmental variables and PAH concentrations in *Rizophora mangle* root tissues during (**A**) the rainy season, (**B**) the dry season. PAHs are classified as low molecular weight (LMW), high molecular weight (HMW), and total PAHs (ΣPAH). Environmental variables include temperature (Temp), salinity (Sal), conductivity (Cond), oxygen saturation (OS), pH, redox potential (Redox), coarse sand (Csand), fine sand (Fsand), and silt. Statistical significance is indicated as p-value < 0.05 (*), < 0.01 (**)

Spearman correlation analysis revealed a significant and positive correlation between silt content (Table 2) and total PAH concentrations (Σ PAH) in *R. mangle* during the rainy season (p-value = 0.033; Fig. 2A).

**Table 2:** Mean values of physicochemical variables (± standard deviation), including temperature (Temp), salinity (Sal), conductivity (Cond), oxygen saturation (OS), dissolved oxygen (DO), pH, redox potential (Redox), coarse sand (Csand), fine sand (Fsand), and silt, by sampling location and season (rainy and dry) in the Ciénaga Grande de Santa Marta (CGSM) and Cispatá Bay (CIS), each with three sampling points

Physicochemical variables	Rainy		Dry	
	CIS	CGSM	CIS	CGSM
Temp (°C)	29.17 ± 1.01	30.48 ± 0.59	30.31 ± 0.16	30.40 ± 2.01
Sal	16.30 ± 2.69	1.76 ± 0.25	30.54 ± 0.79	21.90 ± 3.98
Cond (mS cm^−1^)	29.06 ± 4.93	3.82 ± 0.57	53.84 ± 1.83	38.56 ± 6.01
OS (%)	60.90 ± 5.67	86.73 ± 11.41	84.94 ± 7.71	90.22 ± 14.95
OD (mg L^−1^)	4.10 ± 0.29	6.44 ± 0.80	5.43 ± 0.43	6.01 ± 0.85
pH	7.54 ± 0.10	8.34 ± 0.40	7.86 ± 0.15	8.35 ± 0.16
Redox (mV)	71.67 ± 11.68	58.33 ± 16.80	32.33 ± 0.58	54.33 ± 18.04
Csand (g)	3.81 ± 3.55	20.99 ± 10.05	6.40 ± 5.56	2.56 ± 3.64
Fsand (g)	18.15 ± 17.14	38.26 ± 18.03	25.95 ± 3.41	28.27 ± 4.42
Silt (g)	78.04 ± 15.61	40.75 ± 19.33	67.64 ± 7.38	69.17 ± 7.51
OM (g)	0.46 ± 0.44	0.34 ± 0.31	0.35 ± 0.07	0.23 ± 0.11

No significant correlations were observed during the dry season (Fig. 2B). Nonetheless, the predominance of HMW compounds in total PAH loads was consistent across both seasons.

**Fig. 3:**
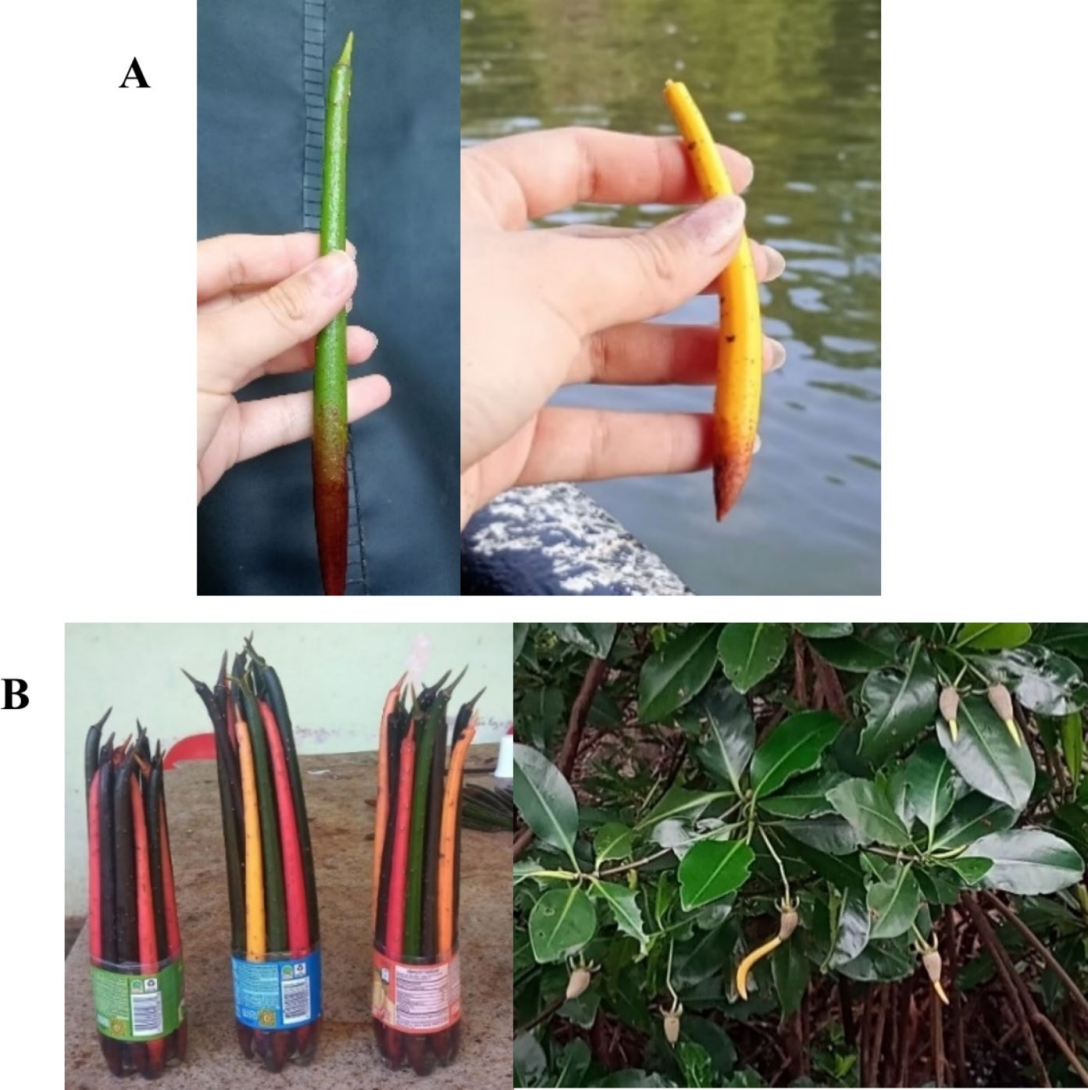
Photographic record of *Rizophora mangle* propagule collection in Cispatá Bay, Colombian Caribbean. (**A**) Comparison between normally pigmented propagules and albino propagules. (**B**) Visual evidence of altered chlorophyll concentrations, indicated by brown, reddish, and yellow pigmentation in propagules

The average Biota-Sediment Accumulation Factor (BSAF) for PAHs during the rainy season was 0.8 ± 0.3 in CGSM (ranging from 0.5 to 1.0) and 0.9 ± 0.6 in CIS (ranging from 0.6 to 1.5), with similar contributions from compounds such as Ch, BbF, and BkF. In the dry season, BSAF values decreased markedly, averaging 0.5 ± 0.1 in CGSM (ranging from 0.4 to 0.6) and 0.2 ± 0.2 in CIS (ranging from 0.04 to 0.4), indicating reduced accumulation during this period. Moreover, symptoms of chlorophyll deficiency and albinism were observed *R. mangle* individuals at two sampling locations within Cispatá Bay (Fig. 3). These phenotypic anomalies were recorded near sites CIS-1 and CIS-3 (see Fig. 1).

### *PCB concentrations*

The highest concentrations of total PCBs in mangrove roots were recorded in the CGSM during the dry season, with an average of 10.7 ± 11.6 ng g^−1^ (ranging from 9.0 to 23.0 ng g^−1^). Five compounds were detected: PCB 28, PCB 52, PCB 101, PCB 138, and PCB 180. Light congeners (e.g., PCB 28, PCB 52, and PCB 101) predominated in this location and season, while heavy PCBs constituted approximately 25.0% of the total load (Table 3). In the same location during the rainy season, lower concentrations were recorded averaging 1.2 ± 2.0 ng g^−1^ (with a single detection of 3.5 ng g^−1^), and only congeners present (e.g., PCB 180).

**Table 3:** Mean PCB concentrations (± standard deviations; ng g⁻^1^ dry weight) in *Rhizophora mangle* root tissues by sampling location, in the Ciénaga Grande de Santa Marta (CGSM) and the Cispatá Bay (CIS), each with three sampling points, by climatic season (rainy and dry), and by individual PCB congeners

Compound	Rainy		Dry	
	CIS	CGSM	CIS	CGSM
PCB 28	2.1 ± 3.6	ND	3 ± 5.2	5.3 ± 4.7
PCB 52	1.4 ± 2.4	ND	ND	1.3 ± 2.3
PCB 101	ND	ND	ND	1.3 ± 2.3
PCB 138	2.9 ± 2.5	ND	ND	1.3 ± 2.3
PCB 180	2.5 ± 2.2	1.2 ± 2.0	ND	1.3 ± 2.3
Light PCBs	3.5 ± 5.9	ND	3 ± 5.2	8 ± 7.6
Heavy PCBs	5.4 ± 2.4	1.2 ± 2.0	ND	2.7 ± 2.3
% Heavy PCBs	60.9 ± 25.0	100	ND	25.0 ± 10.0
ΣPCB	8.9 ± 9.5	1.2 ± 2.0	3 ± 5.2	10.7 ± 11.6

* All congeners belong to Group 2 A (‘probably carcinogenic to humans’) according to IARC (2015). Light PCBs: 28, 52, 101, 118. Heavy PCBs: 138, 153, 180. ND: not detected (values below detection limits)

In CIS, the highest PCB concentration occurred during the rainy season, averaging 8.9 + 9.5 ng g^−1^ dw (range: 7.6–18.9 ng g^−1^). These values were dominated by heavy PCBs (PCB 138 and PCB 180), which together accounted for approximately 60.9% of the total PCB concentrations (Table 3). In contrast, during the dry season, only PCB 28 was detected in CIS, with a mean concentration of 3.0 + 5.2 ng g^−1^ in *R. mangle* roots.

**Fig. 4:**
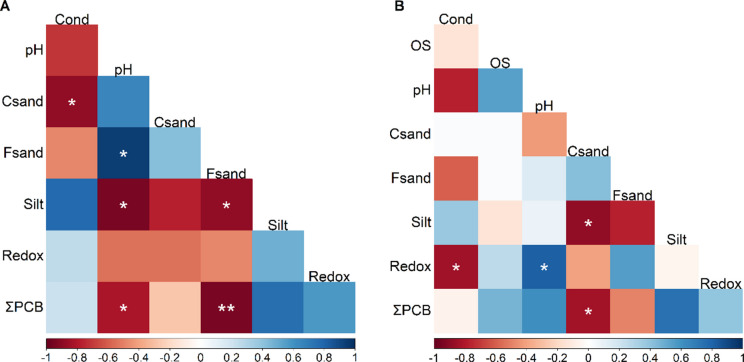
Spearman correlation matrices between environmental variables and total PCB concentrations (ΣPCBs) in *Rizophora mangle* root tissues during (**A**) the rainy season and (**B**) the dry season. Environmental variables include salinity (Sal), conductivity (Cond), oxygen saturation (OS), pH, redox potential (Redox), coarse sand (Csand), fine sand (Fsand), and silt. Statistical significance is indicated as p-values < 0.05 (*), < 0.01 (**)

Spearman correlation analysis (Fig. 4) revealed a strong negative correlation during the rainy season between total PCB concentrations and fine sand (Fsand, Table 2) (p-value = 0.005), as well as a weak negative correlation with pH (p-value = 0.046). In the dry season, a significant negative correlation was observed between total PCB concentrations and coarse sand (Csand; Table 2) (p-value = 0.039).

## Discussion

### *PAH concentrations inR. mangletissues*

The highest PAH concentrations in mangrove roots were recorded in Cispatá Bay (CIS). During the rainy season, high molecular weight (HMW) and carcinogenic (PAH^C^) compounds predominated, whereas in the dry season both LMW and HMW compounds were present, with fewer PAH^C^. This seasonal increase, particularly during the rainy season, likely reflects intensified surface runoff and hydrological flow (Burgos-Núñez et al., 2017; Caballero-Gallardo et al., 2021). The predominance of HMW PAHs such as Ch, Py, BbF, and BkF, suggests high environmental availability and strong retention in *R. mangle* root tissues (Li et al., 2015; Zhang et al., 2017). Notably, the presence of PAH^C^ poses potential long-term risks due to their mutagenic effects and tendency to bioaccumulate (Burgos-Núñez et al., 2017; Proffitt & Travis, 2005; Veldkornet et al., 2020).

Seasonal fluctuations in the relative abundance of LMW and HMW compounds, particularly the increase in LMW PAHs during the dry season, may reflect nearby sources of incomplete combustion (Burgess et al., 2003; Dudhagara et al., 2016; Li et al., 2014). In contrast, the Ciénaga Grande de Santa Marta (CGSM) exhibited lower overall PAH concentrations, with LMW and HMW compounds detected during the rainy season, and HMW and PAH^C^ (50%) predominating in the dry season. This pattern points to a reduced environmental availability of PAHs in CGSM compared to CIS, although associated risks persist, particularly due to accumulation of mutagenic compounds such as Ch, BbF, and BkF within sediments (Behera et al., 2018; Li et al., 2015; Meng et al., 2019; Proffitt & Travis, 2005; Veldkornet et al., 2020).

Comparisons with international studies show that PAH concentration in *R. mangle* roots are within, the range reported for *Avicennia marina* in Saudi Arabia (1.11 to 30.96 ng g^−1^; El-Amin Bashir et al., 2017). But below the elevated values reported in Chinese mangrove ecosystems, (e.g.,440 to 6882 ng g^−1^in Shenzhen) and 368 to 552 ng g^−1^on Hainan Island; Li et al., 2014; Qiu et al., 2018). These differences may reflect higher pollution levels abroad and species-specific variation in PAH accumulation (Kathiresan & Bingham, 2001; Qiu et al., 2018).

Considering the seasonal variability in these estuarine areas and predominance of HMW PAHs, a mixed anthropogenic origin is inferred although mainly associated with pyrogenic processes (Sun et al., 2018; Wang et al., 2014). Likely sources include agricultural burning, vehicular traffic, and maritime traffic. However, PAH concentrations detected in root tissues are also regulated by specific biological characteristics of *R. mangle*, the physicochemical properties of the tissue analyzed, and local environmental conditions that facilitate the adsorption of hydrophobic compounds (Duke & Watkinson, 2002; Naidoo & Naidoo, 2018; Veldkornet et al., 2020). The detection of PAHs in other organisms within CIS, such as fish and birds (Burgos-Núñez et al., 2017), highlights the systemic nature of contamination in this ecosystem.

PAH concentrations are often higher in leaves compared to other tissues such as branches or roots, possibly due to atmospheric deposition or internal translocation (Li et al., 2014; Qiu et al., 2018). Furthermore, the consistent detection of phenanthrene across all sampling points and seasons underscores the affinity of root tissues for specific compounds and their widespread bioavailability (e.g., El-Amin Bashir et al., 2017; Qiu et al., 2018; Veldkornet et al., 2020).

Identifying PAH sources in mangrove systems is complex due to their distribution among multiple ecosystem compartments, particularly sediments (Burgess et al., 2003; Sarria-Villa et al., 2016; Behera et al., 2018; Meng et al., 2019). Nonetheless, combustion-related origins are strongly suggested (Sun et al., 2018; Wang et al., 2014). Effective mitigation requires integrated strategies, including improved wastewater treatment and reductions in combustion-derived emissions (Abdel-Shafy & Mansour, 2016; Li et al., 2014; Mojiri et al., 2019).

### *Indexes and correlations with physicochemical parameters*

Positive correlations between HMW PAHs and particle size support the hypothesis of sediment-mediated absorption (Li et al., 2014; Qiu et al., 2018). *R. mangle* roots serve as primary reservoirs for HMW compounds (Naidoo & Naidoo, 2016), while LMW compounds tend to be more readily transferred to fruits and leaves (Qiu et al., 2018; Veldkornet et al., 2020).

BSAF values indicate low bioaccumulation efficiency in roots tissues. However, the consistent detection of compounds such as Ch, BbF, and BkF, which have been associated with chlorophyll deficiency and albinism, suggests potential sublethal effects due to chronic contamination (Duke & Watkinson, 2002; Proffitt & Travis, 2005; Veldkornet et al., 2020).

### *PCB concentrations*

The highest concentrations of PCBs were recorded in Cispatá Bay during the rainy season, dominated by heavy congeners (e.g., PCB 138 and PCB 180). In contrast, the dry season showed a reduction in PCBs, with only PCB 28 detected. CGSM exhibited an inverse pattern: higher concentrations of light PCBs during the dry season, and only low levels of PCB 180 in the rainy season.

The detected PCB concentrations fall within ranges previously reported in mangrove ecosystems (1.8–3.1 ng g^−1^ dw in China; 0.3–120 ng g^−1^ dw in Puerto Rico; Alegría et al., 2016; Qiu et al., 2019). These values are considered environmentally low compared to industrialized regions (Qiu et al., 2019; Zhao et al., 2008).

The low concentrations observed may reflect limited environmental availability due to regulatory restrictions, lower local industrial intensity, and a possible predominance of atmospheric deposition over root uptake (Ministerio de Ambiente y Desarrollo Sostenible, 2011; Zhao et al., 2012; Ministerio de Ambiente, Vivienda y Desarrollo Territorial, 2016; Qiu et al., 2019). Multi-tissue assessments are needed to clarify dominant accumulation pathways.

### *Influence of environmental parameters in PCB concentrations*

PCB retention is strongly influenced by sediment grain size and organic matter content, with greater retention during the dry season due to urban proximity and finer sediment (Badea et al., 2014; Borja et al., 2005; Borrell et al., 2019; INVEMAR, 2018). A negative correlation was observed between PCB concentrations and fine sand in the rainy season, and with coarse sand in the dry season, indicating seasonal shifts in sediment transport dynamics in estuarine areas.

PCB degradation is modulated by redox potential and pH (Borja et al., 2005), while root exudates may further enhance microbial breakdown (Zhao et al., 2012). Although national policies have restricted PCB use (Ministerio de Ambiente y Desarrollo Sostenible, 2011;  Ministerio de Ambiente, Vivienda y Desarrollo Territorial, 2016), their persistence in both sites signals ongoing ecological risks, including hormonal and carcinogenic effects (USEPA, 2013; Pino et al., 2016).

This study provides the first integrated baseline for PAHs and PCBs concentrations in mangrove roots across key estuarine ecosystems of the Colombian Caribbean. Although limitations in spatial representativeness remain, underscoring the need for future research with greater sampling density and broader temporal coverage, these findings offer relevant insights given the global scarcity of research on persistent organic pollutants in mangrove tissues. Notably, the detection of PAH^C^ in particular areas highlight potential risks for nearby communities due to their bioaccumulative nature. Overall, the results identify priority areas for environmental management, establish reference levels for long-term monitoring programs, and emphasize the urgent need to implement mitigation strategies in these ecologically significant systems.

## Conclusions

The sustained presence of PAH^C^ throughout the seasons highlights the potential for long-term harmful effects on local populations through direct exposure, tissue retention, and accumulation of mutagenic alterations. Although the concentrations reported in this study are relatively low in highly contaminated ecosystems, they are still within the ranges associated with ecological stress, making the likelihood of long-term harmful effects significant.

A slightly seasonal pattern was identified for both pollutant groups: PAH concentrations peaked during the rainy season, whereas PCBs were more prevalent in the dry season (PCB congeners 28–52–101–138–180). The environmental persistence and bioavailability of HMW PAHs, such as Ch, Py, BbF, and BkF, enable their accumulation in *R. mangle* roots.

Chronic contamination may trigger physiological alterations in mangrove species, including chlorophyll loss, cellular damage, and enzymatic disruptions, potentially affect reproductive capacity and overall functionality of mangrove populations in key habitats such as Cispatá. Effective management strategies, such as stricter regulations, improved water treatment systems, and reduced emissions are essential for mitigating the influx and bioaccumulation of PAHs and PCBs. Given their documented toxicity and endocrine-disrupting properties, minimizing their ecological footprint is crucial to safeguard mangrove health and food web stability.

## Data Availability

The authors declare that the data supporting the findings of this study are available within the paper.
